# Left atrial conduit strain derived from cardiac magnetic resonance is an independent predictor of left ventricular reverse remodeling in patients with nonischemic cardiomyopathy

**DOI:** 10.1186/s12880-023-01162-8

**Published:** 2024-01-02

**Authors:** Ke Chen, Lei Chang, Rong Huang, Ziyan Wang, Dan Mu, Lian Wang

**Affiliations:** 1 Department of Cardiology, Nanjing Drum Tower Hospital, Affiliated Hospital of Medical School, Nanjing University, Nanjing, China; 2 Department of Cardiology, Nanjing Drum Tower Hospital Clinical College of Nanjing Medical University, Nanjing, China; 3 Department of Cardiology, Nanjing Drum Tower Hospital Clinical College of Jiangsu University, Nanjing, China; 4 Department of Radiology, Nanjing Drum Tower Hospital, Affiliated Hospital of Medical School, Nanjing University, Nanjing, China

**Keywords:** Left atrial conduit strain, Cardiac magnetic resonance, Nonischemic cardiomyopathy, Left ventricular reverse remodeling

## Abstract

**Background:**

In some patients with nonischemic cardiomyopathy (NICM), left ventricular (LV) function improves with medical assistance, resulting in left ventricular reverse remodeling (LVRR). However, predictors of LVRR are not fully understood. The left atrium (LA) has been reported as a prognostic predictor in patients with heart failure (HF). The present study aimed to evaluate clinical predictors of LVRR related to LA function on cardiac magnetic resonance (CMR).

**Methods:**

A total of 103 patients with reduced left ventricular ejection fraction (LVEF) were enrolled in this retrospective study between September 2015 and July 2021. CMR parameters, including strain data, were measured in all patients. Echocardiographic data obtained approximately 2 years after enrollment were analyzed to assess LVRR.

**Results:**

LVRR occurred in 46 patients (44.7%) during follow-up. The value of LA conduit strain was higher in the LVRR group than in the non-LVRR group (6.6 [interquartile range (IQR): 5.6–9.3]% versus 5.0 [IQR: 3.0-6.2]%; *p* < 0.001). The multivariate logistic regression analysis showed that LA conduit strain was an independent predictor of LVRR (odds ratio [OR]: 1.216, 95% confidence interval [CI]: 1.050–1.408; *p* = 0.009). The area under the receiver operating characteristic (ROC) curve of the LA conduit strain was 0.746, and the cutoff value was 6.2%. The Kaplan‒Meier analysis revealed that the incidence of adverse cardiac events was significantly lower in patients with LA conduit strain > 6.2% compared to those with ⩽6.2%. (log-rank test, *p* = 0.019).

**Conclusions:**

LA conduit strain derived from CMR is an independent predictor of LVRR in patients with NICM.

**Supplementary Information:**

The online version contains supplementary material available at 10.1186/s12880-023-01162-8.

## Introduction

Heart failure is a complex clinical syndrome with diverse etiologies. It is one of the most common causes of cardiac death and heart transplantation. During disease progression, energetic abnormalities, toxic injury, and inflammation lead to adverse ventricular remodeling. It is characterized by changes in the shape, size, and function of the heart muscle [1]. Fortunately, due to natural recovery and guideline-directed medical treatment (GDMT), these changes are reversible in some patients and are known as left ventricular reverse remodeling (LVRR) [2].

The course of LVRR in HF patients can persist for up to 2 years after initial therapy [3]. However, a significant proportion of patients fail to achieve improvement after optimal treatment. In this case, predicting LVRR could guide decision-making when considering more active treatments, such as implantable cardioverter-defibrillator (ICD), cardiac resynchronization therapy, and even heart transplantation [1]. Moreover, a meta-analysis conducted by Kramer et al. proved that patients with LVRR have a lower mortality rate in the long term [4]. Therefore, identifying predictors of LVRR has great prognostic value.

It is now well established from a variety of studies that younger age, female sex, shorter course of disease, nonischemic cause, and fewer comorbidities are associated with a higher likelihood of LVRR [5, 6]. Recently, increasing evidence has shown that enlarged LA is associated with many cardiovascular diseases from a clinical and prognostic perspective [7]. However, LA enlargement itself could not represent the full function of the LA in the cardiac cycle. The emerging CMR evaluation of atrial strain can overcome the limitations of measuring LA volume alone, facilitating the early detection of some cardiovascular diseases [8]. Previous studies have shown that LA strain on CMR has robust prognostic value and helps improve risk stratification in patients with dilated cardiomyopathy [9]. However, to the best of our knowledge, the relationship between LA strain on CMR and LVRR in patients with NICM is not fully understood. Therefore, this study aimed to assess the clinical predictors of LVRR in association with LA strain on CMR in patients with NICM.

## Methods

### Study population

Patients admitted to the hospital for the first time due to HF between September 2015 and July 2021 were reviewed to determine the factors predicting LVRR. During hospitalization, all patients underwent echocardiography for an initial assessment of cardiac function within 48 h after admission. Patients with HFrEF (heart failure with reduced ejection fraction [LVEF ≤ 40%]) were enrolled in this study. Among the 139 patients, 36 were excluded due to the following exclusion criteria: (1) ischemic cardiomyopathy diagnosed by coronary angiography; (2) baseline LVEF > 40%; (3) severe valvular heart disease; and (4) loss to follow-up. Finally, 103 patients with a reduced LVEF were enrolled in this retrospective study. After discharge, all patients received guideline-directed medical treatment (GDMT). At least 6 months after enrollment, they underwent echocardiography again and the changes in cardiac function and incidence of adverse cardiac events were followed up.

The ethics committee of Nanjing Drum Tower Hospital approved the study protocol. The study was conducted following the ethical guidelines of the Declaration of Helsinki. Each patient provided written informed consent.

### Clinical measurements

Well-trained doctors acquired patients’ clinical parameters, medical history, laboratory data, imaging data, and oral medication use from the electronic medical record system. Body mass index (BMI) was calculated as weight divided by the square of the height. Hypertension was defined as diastolic blood pressure (DBP) ≥ 90 mmHg and/or systolic blood pressure (SBP) ≥ 140 mmHg or a self-reported history of hypertension. Hyperlipidemia was defined as fasting triglyceride (TG) ≥ 150 mg/dL, fasting total cholesterol (TC) ≥ 240 mg/dL, high-density lipoprotein cholesterol (HDL-C) < 40 mg/dL, low-density lipoprotein cholesterol (LDL-C)> 160 mg/dL, or previous history of hyperlipidemia. Diabetes mellitus (DM) was defined as hemoglobin A1c (HbA1c) ≥ 6.5%, fasting plasma glucose (FPG) ≥ 126 mg/dL or a history of diabetes [10].

### Echocardiography

Echocardiography was performed on Philips IE33 ultrasound machine by an experienced sonographer following American Society of Echocardiography guidelines [11]. LVEF, left ventricular end-diastolic diameter (LVEDD), left atrial diameter (LAD), interventricular septal thickness at diastole (IVSTD) and left ventricular posterior wall thickness at diastole (LVPWTD) were measured and LVEF was calculated by Biplane Simpson’s area-length method.

### CMR acquisition and feature tracking analysis

**Fig. 1:**
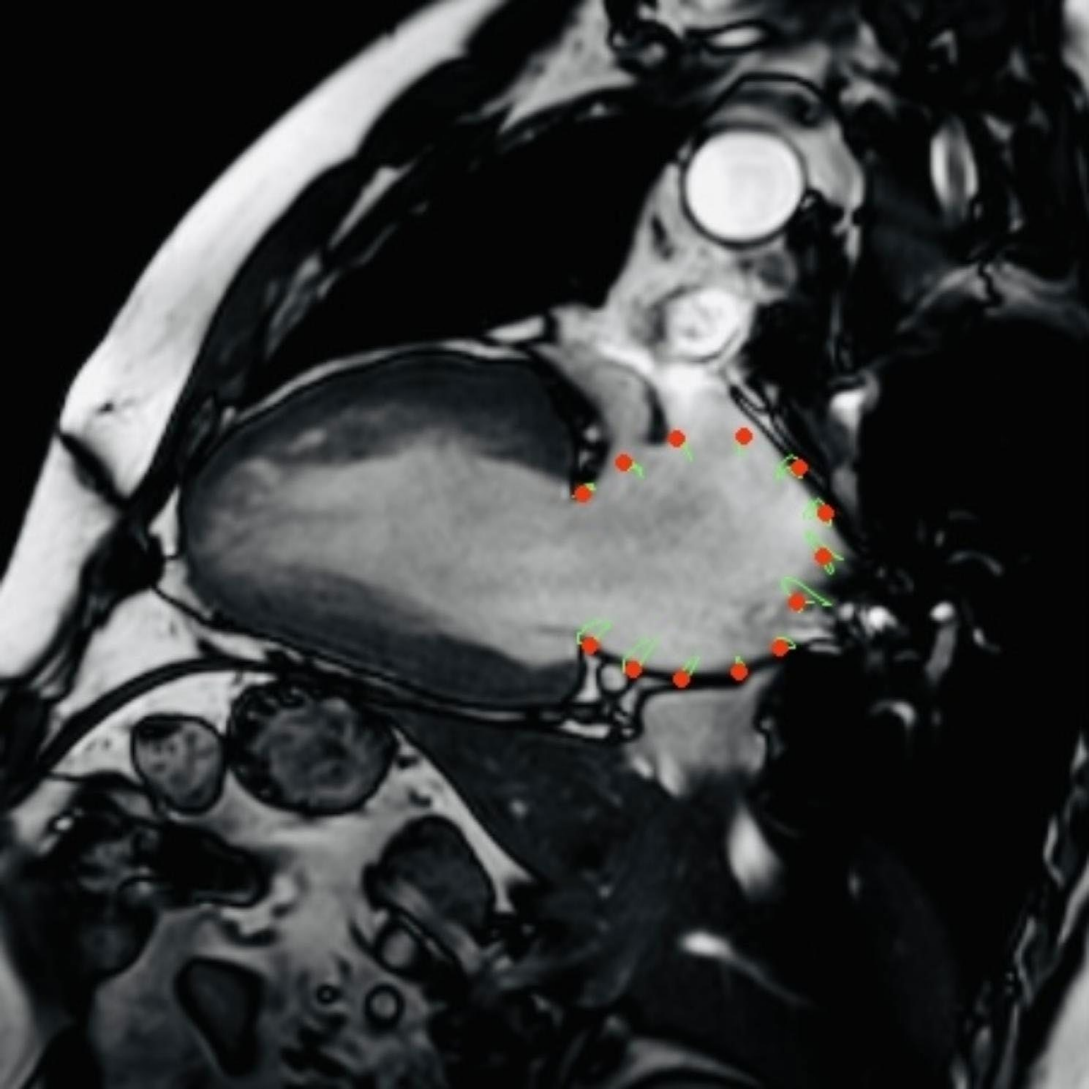
Strain measurement by cardiac magnetic resonance feature tracking. The red dots were used to mark the endocardial contour, and the green curve was used to describe the movement of the target myocardium

All imaging was performed on a 1.5T Philips Achieva CMR scanner (Philips HealthCare, Best, NL, USA) or a 3.0T Ingenia CX CMR scanner (Philips Healthcare, Best, The Netherlands). Electrocardiogram and respiratory gating techniques were required to avoid motion artifacts when acquiring CMR images. Medis software (Medis Medical Imaging Systems) was used for processing and analysis. The imaging modality included two-chamber and four-chamber long-axis views. LA volumes and atrial EF were measured by Biplane Simpson’s area-length method. Under the supervision of a CMR physician with > 5 years of experience and blinded to the results, two cardiac radiologists independently performed feature-tracking strain analysis with the help of Medis Qstrain software (Medis Medical Imaging Systems, version 4.0.24.4). Endocardial contours were automatically drawn and manually adjusted, after which the software was used to track the contours and record the movement of the target myocardium (Fig. 1). Consistent with the measurement method of Scatteia A et al. [12, 13], LA strain was calculated as (L1-L0)×100%/L0, where L0 is the initial length of the myocardial segment at left ventricular end diastole (LVED), and L_1_ is the length at left ventricular end systole (LVES). The left atrium was divided into three segments to measure the left wall, roof, and right wall strain. LA reservoir strain, conduit strain, and booster strain were calculated at late LV systole and early and late LV diastole, respectively. All data obtained from CMR are the average value calculated after two repeated measurements. Inter- and intraobserver variability for LA strain parameters were evaluated by selecting 20 patients randomly and each observer was blinded to the previous result when beginning a new measurement.

### Clinical endpoints

The primary endpoint was the recovery of cardiac function. At least 6 months after enrollment, all patients underwent echocardiography to evaluate the changes in cardiac function. During follow-up, Punnoose et al. identified an increase in LVEF in some patients with previously reduced ejection fraction [14]. To describe and promote the study of characteristics of this distinct clinical entity, relevant heart failure (HF) associations issued the consensus statement to universally define HF with improved ejection fraction (HFimpEF): HF with follow-up measurement of LVEF > 40% and a ≥ 10% increase from previous LVEF of ≤ 40% [15], which was regarded as a more appropriate definition for LVRR in our study. Patients were divided into two groups, i.e., (1) the LVRR group, with a follow-up measurement of LVEF > 40% and a ≥ 10% increase from previous LVEF of ≤ 40%; and (2) the non-LVRR group, with a follow-up measurement of LVEF ≤ 40% or not improved by ≥ 10 points (patients who died of HF within 6 months after enrollment were classified as the non-LVRR group). The time interval of echocardiography examination did not significantly differ between the LVRR group and non-LVRR group (20.5 [11.0-35.3] months vs. 17.0 [8.5–38.0] months; *p* = 0.552). Follow-up methods included outpatient visits, telephone contact with patients, and review of medical records. The secondary endpoints included adverse cardiac events, which were defined as cardiovascular (CV) death or unexpected rehospitalization for worsening HF. The follow-up lasted for a period of 26.0 (15.0–43.0) months.

### Statistical analysis

Frequency (percentage) was used to express categorical variables. For continuous variables, after evaluating the normality of the data by the Shapiro‒Wilk normality test, mean with SD and median [IQR] were used to express data conforming to the normal distribution and those with nonnormal distribution, respectively. The data of the two groups, the LVRR group and the non-LVRR group, were compared by unpaired t test or Mann‒Whitney U test for continuous variables and Pearson chi-square test for categorical variables. Intraclass correlation coefficient (ICC) was used to assess inter- and intraobserver agreement.

LVRR predictors were estimated by univariate and multivariate logistic regression analysis. The variables in the univariate analysis were well-established parameters that may influence the progression of heart failure. The multivariable analysis incorporated all variables univariably significantly related to the outcomes (*P* values < 0.05) and all of these variables were not collinear. Next, predictor selection was performed stepwise in the forward direction, and only predictors with *P* values < 0.05 were displayed in the final clinical model. Odds ratios (ORs) and 95% confidence intervals (CIs) were used to describe the results. Receiver operating characteristic (ROC) analysis was performed to investigate LVRR predictability, and patients were divided into two groups according to the LA conduit strain cutoff value. Kaplan‒Meier curves were constructed to describe the survival status of patients from different groups, and the log-rank test was used for comparison. Spearman’s correlation coefficient was computed to assess the correlation between LA conduit strain and other LA continuous variables.

All data were analyzed with R version 4.2.2 and SPSS for Windows version 26, and a *P* value < 0.05 indicated statistical significance.

**Fig. 2:**
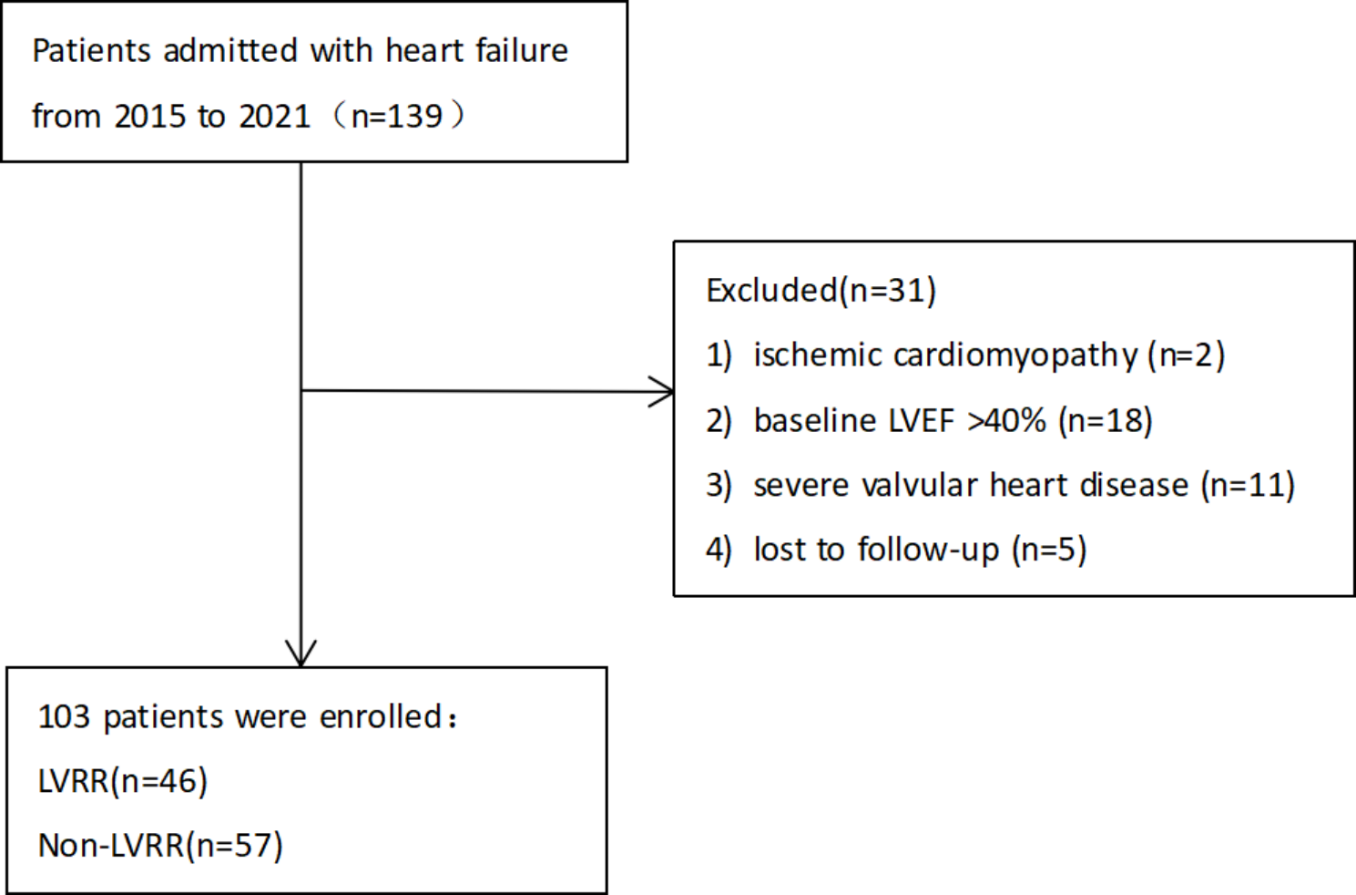
Flowchart of patient selection

## Results

### Patient population

The flowchart of patient selection is shown in Fig. 2. This retrospective study initially reviewed 139 patients with HF. After excluding patients who did not meet the criteria, 103 patients were analyzed. Table 1 summarizes the baseline characteristics of the LVRR and non-LVRR groups. According to the echocardiography results, 46 (44.7%) patients experienced LVRR. There were no significant differences in sex, body mass index (BMI), New York Heart Association (NYHA) functional class, or resting systolic and diastolic blood pressures. Patients with LVRR were younger and had higher heart rates (HRs) than those without LVRR. The presence of medical history and oral medication use were similar between the two groups. No significant differences were observed in fasting plasma glucose, C-reactive protein, hemoglobin or B-type natriuretic peptide, while TG was higher in the LVRR group.

**Table 1:** Baseline characteristics of patients with and without left ventricular reverse remodeling (LVRR)

	LVRR(n = 46)	Non-LVRR(n = 57)	*P* value
Female,n(%)	9(19.6)	14(24.6)	0.545
Age(years)	44.2 ± 14.6	50.2 ± 14.7	0.042
BMI(kg/m^2^)	26.6 ± 4.5	25.5 ± 4.9	0.232
NYHA functional class>II,n(%)	28(60.9)	32(56.1)	0.628
**Medical history, n(%)**			
Hypertension	27(58.7)	30(52.6)	0.538
Hyperlipidemia	10(21.7)	6(10.5)	0.118
Diabetes mellitus	12(26.1)	9(15.8)	0.197
Atrial fibrillation	2(4.3)	9(15.8)	0.122
**Vital singns**			
HR(bpm)	91.8 ± 13.9	83.9 ± 16.4	0.011
SBP(mmHg)	124.3 ± 23.9	123.4 ± 20.9	0.845
DBP(mmHg)	80.5(73.0-96.3)	80.0(67.0–88.0)	0.291
**Treatments, n (%)**			
ACEI/ARB/ARNI	40(87.0)	51(89.5)	0.692
Beta-blocker	41(89.1)	54(94.7)	0.492
Aldosterone antagonist	43(93.5)	55(96.5)	0.806
Loop diuretic	42(91.3)	48(84.2)	0.281
SGLT2i	5(10.9)	3(5.3)	0.492
Digoxin	8(17.4)	15(26.3)	0.280
**Laboratory data**			
TG(mmol/L)	1.4(1.1–1.8)	1.1(0.9–1.6)	0.005
TC(mmo/L)	4.3 ± 0.9	4.2 ± 1.0	0.639
HDL-C(mmol/L)	0.9 ± 0.3	1.0 ± 0.3	0.198
LDL-C(mmo/L)	2.6 ± 0.7	2.6 ± 0.8	0.713
Fasting plasma glucose(mmol/L)	5.0(4.6–6.1)	4.9(4.3–5.5)	0.065
C-reactive protein(mg/L)	3.8(2.7–5.7)	4.2(2.5–5.9)	0.832
Creatinine(μmol/L)	77.7(66.9–85.1)	81.0(59.5–92.7)	0.559
Hb(g/L)	148.4 ± 19.4	144.8 ± 20.4	0.367
BNP(pg/mL)	517.0(308.7-933.8)	672.0(223.0-1160.0)	0.434

Values are the mean ± standard deviation, median (interquartile range), or n (%)

**Table 2:** Baseline echocardiography and CMR parameters

	LVRR(n = 46)	Non-LVRR(n = 57)	*P* value
**Baseline echocardiographic data**			
LVEF(%)	29.5(25.8–32.0)	27.0(24.0-30.5)	0.141
LVEDD(mm)	66.7 ± 6.4	69.7 ± 7.5	0.035
LAD(mm)	47.6 ± 6.2	49.3 ± 6.6	0.209
IVSTD(mm)	8.5(8.0-9.3)	8.1(7.5–9.2)	0.430
LVPWTD(mm)	8.7(7.8–9.5)	8.3(7.6–9.3)	0.444
**Left atrial CMR Parameters**			
LA volume at LVED(ml)	64.55(38.8–98.0)	98.2(57.4-117.1)	0.009
LA volume at LVES(ml)	100.4 ± 41.9	126.7 ± 54.0	0.008
Ejection fraction(%)	31.8(21.4–40.0)	26.2(17.1–34.4)	0.067
Global circumferential strain(%)	13.4(7.5–17.9)	8.6(5.5–17.8)	0.106
Global longitudinal strain			
Reservoir strain(%)	16.3(10.5–20.1)	12.5(7.8–18.0)	0.023
Conduit strain(%)	6.6(5.6–9.3)	5.0(3.0-6.2)	<0.001
Booster strain(%)	8.7(4.9–10.7)	7.0(4.2–13.3)	0.392
Segmental strain			
Left wall strain(%)	18.2(12.3–24.4)	14.6(9.3–24.2)	0.264
Roof strain(%)	11.8(7.3–18.3)	10.0(5.1–16.6)	0.195
Right wall strain(%)	19.7(12.0-25.6)	14.8(9.1–22.6)	0.111

Values are the mean ± standard deviation or median (interquartile range)

### Echocardiography and CMR parameters

Table 2 summarizes the results of echocardiography and CMR parameters in the LVRR group and non-LVRR group. Baseline echocardiographic data showed that the left ventricular end-diastolic diameter (LVEDD) was shorter in the LVRR group, while there was no significant difference in LVEF or left atrial diameter. For left atrial magnetic resonance data, LA volume at LVED and LVES was significantly smaller in the LVRR group. The two groups did not differ in LAEF or left atrial global circumferential strain (LAGCS). The value of left atrial global longitudinal strain (LAGLS), including reservoir and conduit strain, was significantly higher in the LVRR group (reservoir strain: 16.3 [IQR: 10.5–20.1]% versus 12.5 [IQR: 7.8–18.0]%; *p* = 0.023; conduit strain: 6.6 [IQR: 5.6–9.3]% versus 5.0 [IQR: 3.0-6.2]%; p<0.001), while there was no difference in booster strain. Generally, the value of LA segmental strain was higher in patients with LVRR, although the difference was not statistically significant.

### Predictive performance for LVRR

**Fig. 3:**
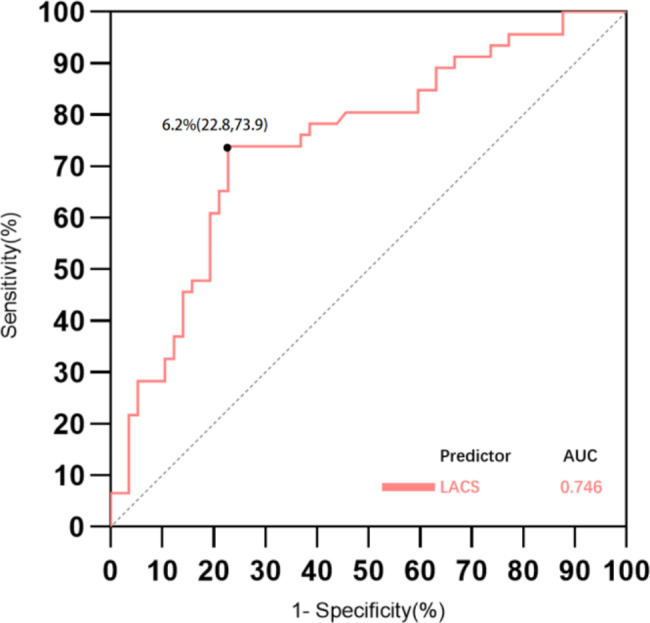
Predictive performance of left atrial conduit strain (LACS) for left ventricular reverse remodeling. Receiver operating characteristic curves of left atrial conduit strain (LACS) to predict left ventricular reverse remodeling

The results of the univariate and multivariate logistic regression analysis are shown in Table 3. In the univariate analysis, age (OR: 0.972, 95% CI: 0.946–0.999; *p* = 0.045), heart rate (OR: 1.035, 95% CI: 1.007–1.063; *p* = 0.013) and triglyceride (OR: 2.516, 95% CI: 1.116–5.668; *p* = 0.026) were significantly associated with LVRR. For left atrium data, LA volume at LVED, LA volume at LVES, and LA conduit strain were all predictors of LVRR. Variables that were univariably associated with LVRR were not collinear and were included in the multivariate analysis (*P* values < 0.05). Adjusted for age, heart rate, triglyceride, LA volume at LVED and LVES, the multivariate logistic regression analysis revealed that LA conduit strain was an independent predictor of LVRR (OR: 1.269, 95% CI: 1.099–1.466; *p* = 0.001). The predictive performance of LA conduit strain for LVRR is shown in Fig. 3. LA conduit strain demonstrated strong predictability for LVRR (area under the curve [AUC] = 0.746) with a cutoff value of 6.2%, providing 77.2% specificity and 73.9% sensitivity.

**Table 3:** Univariate and multivariate analysis for LVRR

		Univariate analysis				Multivariate analysis			
Variables		OR	95%CI	*P* value		OR	95%CI		*P* value
Female		0.747	0.290–1.924	0.546					
Age(years)		0.972	0.946–0.999	0.045					
BMI(kg/m2)		1.053	0.968–1.146	0.231					
HR(bpm)		1.035	1.007–1.063	0.013		1.047	1.015–1.080		0.004
SBP(mmHg)		1.002	0.984–1.020	0.843					
DBP(mmHg)		1.019	0.994–1.045	0.129					
Hypertension		1.279	0.584–2.801	0.539					
Hyperlipidemia		2.361	0.787–7.082	0.125					
Diabetes mellitus		1.882	0.714–4.963	0.201					
Atrial fibrillation		0.242	0.050–1.184	0.080					
ACEI/ARB/ARNI		0.784	0.235–2.617	0.693					
Beta-blocker		0.456	0.103–2.017	0.300					
Aldosterone antagonist		0.521	0.083–3.259	0.486					
SGLT2i		2.195	0.496–9.719	0.300					
Digoxin		0.589	0.225–1.545	0.282					
TG		2.516	1.116–5.668	0.026					
TC		1.107	0.727–1.685	0.635					
HDL-C		0.429	0.114–1.623	0.213					
LDL-C		1.105	0.653–1.869	0.710					
Fasting plasma glucose(mmol/L)		1.167	0.890–1.529	0.264					
C-reactive protein(mg/L)		1.005	0.977–1.035	0.717					
Creatinine(μmol/L)		1.003	0.993–1.013	0.578					
Hb(g/L)		1.009	0.989–1.029	0.364					
log BNP		0.972	0.476–1.988	0.939					
LV ejection fraction(%)		1.036	0.961–1.116	0.359					
LA volume at LVED(ml)		0.988	0.979–0.998	0.015					
LA volume at LVES(ml)		0.988	0.980–0.997	0.011					
LA ejection fraction(%)		1.021	0.993–1.051	0.142					
LA global circumferential strain(%)		1.020	0.980–1.062	0.329					
LA reservoir strain(%)		1.036	0.993–1.081	0.098					
LA conduit strain(%)		1.231	1.072–1.415	0.003		1.269	1.099–1.466		0.001
LA booster strain(%)		1.002	0.950–1.057	0.937					
LA left wall strain(%)		1.019	0.986–1.052	0.262					
LA roof strain(%)		1.001	0.973–1.030	0.941					
LA right wall strain(%)		1.030	0.996–1.067	0.087					

### Associations among LVRR, LA conduit strain, and adverse cardiac events

**Fig. 4:**
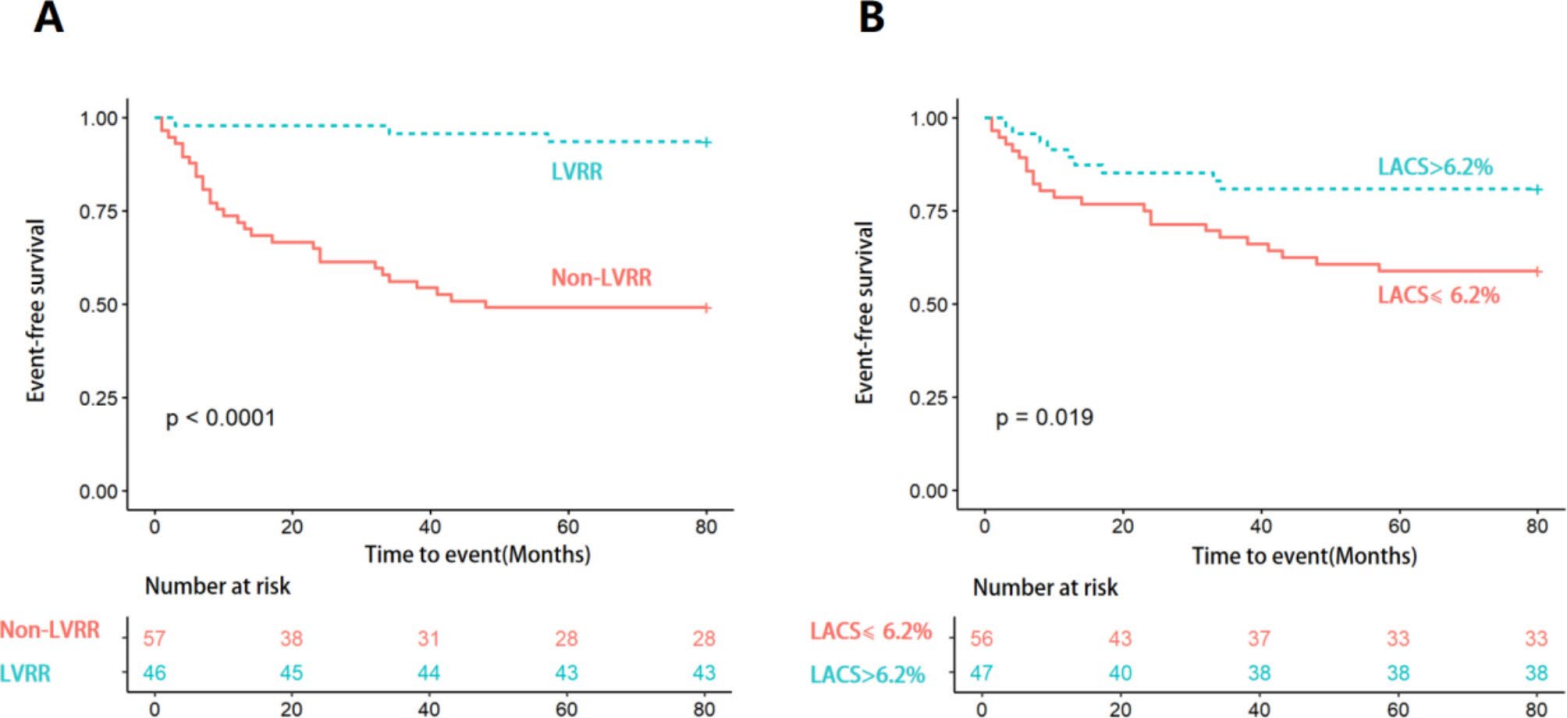
Kaplan‒Meier analysis of the risk of adverse cardiac events. Cardiovascular death or unexpected rehospitalization for worsening HF were significantly less frequent in the LVRR group (**A**; log-rank test, *p* < 0.001) and the LACS > 6.2% group (**B**; log-rank test, *p* = 0.019)

Secondary endpoint events occurred in 4 (8.7%, 4 patients experienced unexpected rehospitalization for worsening HF and no one experienced CV death) patients in the LVRR group and 28 (49.1%, 23 patients experienced unexpected rehospitalization for worsening HF and 5 patients experienced CV death) patients in the non-LVRR group. The unadjusted survival curve showed that patients in the LVRR group had higher event-free survival rates than those in the non-LVRR group (log-rank test, *p* < 0.001; Fig. 4A). Next, patients were divided into two groups according to the LA conduit strain cutoff value, i.e., the left atrial conduit strain (LACS) > 6.2% group and the LACS ≤ 6.2% group. Adverse cardiac events were observed in 9 (19.6%, 8 patients experienced unexpected rehospitalization for worsening HF and 1 patients experienced CV death) patients in the LACS > 6.2% group and 23 (40.4%, 19 patients experienced unexpected rehospitalization for worsening HF and 4 patients experienced CV death) patients in the LACS ≤ 6.2% group. The unadjusted Kaplan–Meier analysis showed that CV death or unexpected rehospitalization for worsening HF was significantly less frequent in the LACS > 6.2% group (log-rank test, *p* = 0.019; Fig. 4B). In total, only five events of CV death were noted during follow-up. Significant differences could not be demonstrated due to the small number of events, but four of the five were observed in the LACS ≤ 6.2% group.

### Correlation between LA conduit strain and other LA continuous variables

The correlations between LA conduit strain and other continuous variables are shown in Table 4. LA conduit strain was positively correlated with LA ejection fraction (Spearman’s r = 0.559, *p* < 0.001), LA right wall strain (Spearman’s r = 0.514, *p* < 0.001), and LA global circumferential strain (Spearman’s r = 0.490, *p* < 0.001) and negatively correlated with LA volume at LVED (Spearman’s r = -0.515, *p* < 0.001) and LA volume at LVES (Spearman’s r = -0.444, *p* < 0.001).

**Table 4:** Correlation between LA conduit strain and other LA continuous variables

	Spearman r	*P* value
LA volume at LVED(ml)	-0.515	< 0.001
LA volume at LVES(ml)	-0.444	< 0.001
LA ejection fraction(%)	0.559	< 0.001
LA global circumferential strain(%)	0.490	< 0.001
LA left wall strain(%)	0.448	< 0.001
LA roof strain(%)	0.441	< 0.001
LA right wall strain(%)	0.514	< 0.001

### Reproducibility analysis

LA strain measurement on CMR showed excellent reproducibility. The ICCs for inter and intraobserver agreement were all > 0.9 for LA strain. The detailed results are shown in supplementary Table 1.

## Discussion

In recent years, a renewed interest in LVRR in patients with HF has been observed. Our Kaplan–Meier analysis suggested that patients with LVRR had a better prognosis. Thus, the prediction of LVRR has an important role in evaluating the severity of HF and carrying out medical intervention in advance to improve the prognosis. Herein, we evaluated the impact of CMR-derived LA strain in predicting LVRR, finding that LA conduit strain was an independent predictor of LVRR. Moreover, LA conduit strain was instrumental in predicting the risk of CV death or unexpected rehospitalization for worsening HF. To the best of our knowledge, this is the first study to demonstrate the association between LA conduit strain derived from CMR and LVRR in patients with NICM.

The left atrium (LA) is a crucial chamber affecting cardiac performance, and its principal function can be divided into three parts: (1) reservoir function, i.e., collecting blood from pulmonary veins during ventricular systole; (2) conduit function, i.e., transferring blood from the LA to the left ventricle (LV) during early LV diastole; and (3) booster function, i.e., promoting ventricular filling by contracting the LA during late diastole [16]. In patients with HF, LV filling pressure is higher than that of healthy people, which reduces the pressure gradient between the LA and LV during early diastole (conduit strain) [17]. Consequently, an increase in LA volume and pressure is inevitable, weakening left atrial compliance (reservoir strain) and damaging systolic function (booster strain) over time [9]. Therefore, it is reasonable to speculate that LA conduit strain may be an early sensor of LV function changes. The excellent predictive value of LA conduit strain for LVRR in our study also demonstrated its strong sensitivity to changes in cardiac function.

Tissue tracking technology is a method of identifying special patterns along a curve on one image and identifying the same pattern in a second image taken seconds later. In this way, the displacement of myocardial segments can be calculated [18]. In echocardiography, the image is characterized by the presence of spots with a certain persistence [19, 20]. In CMR, tissue regions are identified based on individual anatomical features, often regarded as feature tracking (FT) [21]. Therefore, the essential difference between these two techniques relates to the myocardial area monitored, which may lead to differences in measurement results [18]. Assessing LA function using speckle tracking echocardiography(STE) is a relatively simple tool that can reveal the pathophysiological mechanisms underlying a variety of cardiovascular diseases [22]. Recent studies have shown that STE is a feasible method for assessing left atrial deformation and is now the standard method for assessing LA strain [23]. However, STE technology is limited by the low spatial resolution of ultrasound imaging, poor acoustic windows, and operator skills, requiring real-time processing. Therefore, the accuracy and convenience of myocardial strain measurement still need to be improved [24]. CMR represents the gold standard for imaging to assess cardiac geometry and function [8, 25, 26]. Compared with STE, CMR offers a broader view range, higher spatial resolution, better imaging quality, and lower inter- and intraobserver variability [27]. The result of LA strain measurement in our study also highlights the high reproducibility and low observer variability of CMR technique. In addition, CMR feature tracking not only reflects the overall impairment of cardiac function but is also sensitive to local myocardial movement abnormalities. Therefore, LA strain may better reflect the left atrial’s global and local function compared to traditional volumetric parameters [28]. By tracking left atrial function, it is also possible to indirectly evaluate left ventricular diastolic function. Left atrial conduit function and booster function correspond to left ventricular diastolic function in early and late diastole, respectively, breaking the limitations of magnetic resonance imaging in assessing cardiac diastolic function and providing early evidence for clinical diagnosis and intervention [29].

Some studies have revealed that, even without LA volume changes, LA strain can reflect the degree of left ventricular dysfunction [30, 31]. In the present study, the LVRR group had a significantly smaller LA volume and better conduit strain than the non-LVRR group. However, there was no association between LVRR and LA volume in the multivariable analysis. Considering the negative correlation between LA conduit strain and LA volume, it can be inferred that the difference in LA volume was associated with LA conduit strain. In other words, LA volume is not an independent predictor of LVRR. Instead, LA conduit strain measured from CMR can more comprehensively evaluate the state of cardiac function to predict LVRR independently. Previous studies have reported that impaired LA conduit strain derived from CMR is associated with more frequent adverse clinical events [9, 32]. The present study demonstrated that NICM patients with higher values of LA conduit strain were at a lower risk of CV death or unexpected rehospitalization for worsening HF, meaning that LA conduit strain has great prognostic value while predicting LVRR.

Although many current therapies have a certain effect on reversing left ventricular remodeling, the clinical course of HF is quite variable. Improvement in LVEF does not always persist, and deterioration of cardiac function can occur over time. Our results showed that if the LA conduit strain was seriously damaged in NICM patients with reduced LVEF, subsequent cardiac function recovery was less likely. Therefore, LA conduit strain derived from CMR is instrumental in improving prognosis by identifying the degree of cardiac dysfunction and providing early medical assistance in patients with NICM. More studies are needed to establish the importance of LA conduit strain and explore its value in therapy guidance.

### Study limitations

There are several limitations to the present study. First, our study population included patients with NICM with a reduced LVEF. Therefore, our findings might have lower generalizability for patients with HFmrEF (heart failure with mildly reduced ejection fraction) and HFpEF (heart failure with preserved ejection fraction) [33]. Second, patients’ medication changes and treatment compliance may affect the recovery of cardiac function and the occurrence of adverse cardiac events during the follow-up, which inevitably reduces the accuracy of the results. Third, as a single-center prospective study, the number of patients was insufficient to make the results applicable to the general population.

## Conclusion

LA conduit strain derived from CMR is an independent predictor of LVRR in patients with NICM.

### Electronic supplementary material

Below is the link to the electronic supplementary material.

**Supplementary Material 1: Supplementary Table 1**. Inter- and intraobserver agreement for LA strain

## Data Availability

Information and data concerning the study population were extracted from hospital information systems. To protect the privacy of participants, this dataset is not made public. However, if your request is reasonable, data could be obtained from the corresponding author.

## Abbreviations

ACEI: Angiotensin-converting enzyme inhibitor
ARB: Angiotensin receptor inhibitor
ARNI: Angiotensin receptor neprilysin inhibitor
AUC: Area under the curve
BMI: Body mass index
BNP: B-type natriuretic peptide
CI: Confidence interval
CMR: Cardiac magnetic resonance
CV: Cardiovascular
DBP: Diastolic blood pressure
DM: Diabetes mellitus
GDMT: Guideline-directed medical treatment
Hb: Hemoglobin
HbA1c: Hemoglobin A1c
HDL-C: High-density lipoprotein cholesterol
HF: Heart failure
HFimpEF: Heart failure with improved ejection fraction
HFmrEF: Heart failure with mildly reduced ejection fraction
HFpEF: Heart failure with preserved ejection fraction
HFrEF: Heart failure with reduced ejection fraction
HR: Heart rate
ICC: Intraclass correlation coefficient
ICD: Implantable cardioverter-defibrillator
IQR: Interquartile range
IVSTD: Interventricular septal thickness at diastole
kg: Kilogram
LA: Left atrium/atrial
LACS: Left atrial conduit strain
LAD: Left atrial diameter
LDL-C: Low-density lipoprotein-C
LV: Left ventricle/ventricular
LVED: Left ventricular end diastole
LVEDD: Left ventricular end-diastolic diameter
LVEF: Left ventricular ejection fraction
LVES: Left ventricular end systole
LVPWTD: Left ventricular posterior wall thickness at diastole
LVRR: Left ventricular reverse remodeling
m: Meter
NICM: Nonischemic cardiomyopathy
NYHA: New York Heart Association
OR: Odds ratio
ROC: Receiver operating characteristic
SBP: Systolic blood pressure
SD: Standard deviation
SGLT2i: Sodium-glucose cotransporter 2 inhibitor
STE: Speckle tracking echocardiography
TC: Total cholesterol
TG: Triglyceride
