# Supplementary material for: Left atrial conduit strain derived from cardiac magnetic resonance is an independent predictor of left ventricular reverse remodeling in patients with nonischemic cardiomyopathy
Source: BMC Med Imaging. 2024 Jan 2;24:2. doi: 10.1186/s12880-023-01162-8 (PMC10759573; doi:10.1186/s12880-023-01162-8)
Supplement: Supplementary file 1 — Supplementary Material 1: Supplementary Table 1. Inter- and intraobserver agreement for LA strain [file 12880_2023_1162_MOESM1_ESM.doc]

Supplementary Table 1. Inter- and intraobserver agreement for LA strain

|  | Interobserver agreement | | | Intraobserver agreemet | | |
| --- | --- | --- | --- | --- | --- | --- |
|  | ICC | 95%CI | P value | ICC | 95%CI | P value |
| Global circumferential strain(%) | 0.978 | 0.946-0.991 | ＜0.001 | 0.994 | 0.983-0.998 | ＜0.001 |
| Global longitudinal strain |  |  |  |  |  |  |
| Reservoir strain(%) | 0.971 | 0.925-0.989 | ＜0.001 | 0.993 | 0.984-0.997 | ＜0.001 |
| Conduit strain(%) | 0.920 | 0.810-0.968 | ＜0.001 | 0.974 | 0.933-0.990 | ＜0.001 |
| Booster strain(%) | 0.952 | 0.885-0.981 | ＜0.001 | 0.981 | 0.953-0.992 | ＜0.001 |
| Segmental strain |  |  |  |  |  |  |
| Left wall strain(%) | 0.979 | 0.948-0.992 | ＜0.001 | 0.988 | 0.845-0.997 | ＜0.001 |
| Roof strain(%) | 0.983 | 0.958-0.993 | ＜0.001 | 0.983 | 0.959-0.993 | ＜0.001 |
| Right wall strain(%) | 0.941 | 0.859-0.976 | ＜0.001 | 0.968 | 0.921-0.987 | ＜0.001 |
